# A Single Common Assay for Robust and Rapid Fragile X Mental Retardation Syndrome Screening From Dried Blood Spots

**DOI:** 10.3389/fgene.2018.00582

**Published:** 2018-11-27

**Authors:** Vivienne J. Tan, Mulias Lian, Sultana M.H. Faradz, Tri I. Winarni, Samuel S. Chong

**Affiliations:** ^1^Department of Paediatrics, Yong Loo Lin School of Medicine, National University of Singapore, Singapore, Singapore; ^2^Khoo Teck Puat – National University Children’s Medical Institute, National University Health System, Singapore, Singapore; ^3^Division of Human Genetics, Center for Biomedical Research, Faculty of Medicine, Diponegoro University, Semarang, Indonesia; ^4^Department of Laboratory Medicine, National University Hospital, National University Health System, Singapore, Singapore

**Keywords:** fragile X syndrome, *FMR1*, trinucleotide repeat, dried blood spot, triplet-primed PCR (TP-PCR), melt curve analysis (MCA)

## Abstract

**Background:** *FMR1* CGG trinucleotide repeat hyper-expansions are observed in 99% of individuals with fragile X mental retardation syndrome (FXS). We evaluated the reliability of a rapid single-step gender-neutral molecular screen for FXS when performed on DNA isolated from dried blood spots.

**Methods:** DNA was extracted from dried blood spots of 151 individuals with intellectual disability or autism spectrum disorder, whose *FMR1* repeat genotypes are known. Dried blood spots were blinded prior to DNA extraction and analysis by triplet primed PCR (TP-PCR) and melt curve analysis (MCA). All expansion-positive and representative expansion-negative samples were also genotyped by fluorescent TP-PCR and capillary electrophoresis (CE) to confirm repeat expansion status.

**Results:** Three males and 12 females were classified as expanded by TP-PCR MCA, and were subsequently sized by fluorescent TP-PCR CE. Two males and four females carried premutations, while one male and eight females carried full mutations. All 19 non-expanded samples that were sized were confirmed as carrying only normal alleles. Replicate analysis of representative expansion-positive samples yielded reproducible melt peak profiles. TP-PCR MCA classifications were completely concordant with *FMR1* CGG repeat genotypes.

**Conclusion:** TP-PCR MCA of dried blood spot DNA accurately and reliably identifies presence/absence of *FMR1* CGG repeat expansions in both genders simultaneously. This strategy may be suitable for rapid high-throughput first-tier screening for fragile X syndrome.

## Introduction

Fragile X syndrome (FXS; OMIM 309550) is a leading genetic cause of intellectual disability and autism spectrum disorders. Clinical manifestations include neuro-developmental delay, anxiety, mild to severe intellectual impairment, and macroorchidism in prepubertal males (McLennan et al., 2011). FXS generally affects one in 4000 males and one in 2000–8000 females (Monaghan et al., 2013).

Ninety-nine percent of all FXS cases are caused by aberrant hypermethylation and hyperexpansion of a trinucleotide (CGG) repeat in the 5′ untranslated region of the *FMR1* gene (Pieretti et al., 1991; Sutcliffe et al., 1992) located on chromosome Xq27.3 (Fu et al., 1991; Verkerk et al., 1991). The trinucleotide repeat in most normal (NL) individuals ranges from 5 to 44 repeats, although some individuals may carry intermediate or gray zone (IM/GZ) alleles of 45 to 54 repeats. Individuals with expanded alleles of 55 to 199 repeats are considered premutation (PM) carriers, while the presence of 200 or more repeats, almost always accompanied by methylation of the CpG dinucleotides, is indicative of a full mutation (FM) (Kronquist et al., 2008). Generally, clinical manifestation of FXS only occurs in carriers of methylated FM alleles.

The PM allele is meiotically unstable, especially when it lacks AGG triplet interruptions, resulting in increased risk of expansion to a FM allele when transmitted from females to offspring (Fernandez-Carvajal et al., 2009). This risk increases with PM allele size, reaching 90% when the PM allele is > 90 repeats (Nolin et al., 2003, 2011) and > 98% if the PM allele is > 100 repeats (Nolin et al., 2011). Some PM carriers may also display mild developmental delay and behavioral issues (Bailey, 2004). Moreover, a significant fraction of PM carriers (40% males and 8–16% females) are affected by late-onset fragile X-associated tremor / ataxia syndrome (FXTAS; OMIM 300623) (Jacquemont et al., 2004; Rodriguez-Revenga et al., 2009). Another late-onset condition, fragile X-associated premature ovarian insufficiency (FXPOI; OMIM 311360) afflicts 20% of female PM carriers (Rodriguez-Revenga et al., 2009; Hoyos and Thakur, 2017).

Currently, molecular testing for FXS is only introduced to individuals with a family history of FXS and associated diseases, or individuals with clinical features suggesting FXS, FXTAS, FXPOI or intellectual disability (ACOG, 2017). Lately, there has been increased interest in early detection in conjunction with intervention for FXS. Early detection of FXS before the formative years could allow for more effective intervention and minimization of a family’s diagnostic odyssey (Bailey, 2004). Furthermore, recent studies have shown promise in the treatment of FXS particularly in early childhood (Castagnola et al., 2017; Ligsay et al., 2017). Early detection of PM carrier status allows appropriate counseling on the risks of FXS transmission, and effects of FXPOI or FXTAS, for better family planning and psychological preparedness (Abrams et al., 2012; Tassone, 2014; Hagerman et al., 2017). As the combined population prevalence of FM and PM carriers is less than 1% (Hagerman et al., 2017), there has been recent interest in developing cost-effective and rapid screening strategies to identify PM and FM carriers.

Dried blood spots were previously shown to be suitable DNA sources for large-scale FXS screening of boys with mental retardation using a regular repeat-spanning PCR method (Tzeng et al., 2001; Chow et al., 2003). Tassone et al. later demonstrated that triplet-primed PCR (TP-PCR) and capillary electrophoresis was a sensitive method for second-tier identification of FXS from dried blood spot-derived DNA after a first-tier regular PCR to identify and exclude the majority normal samples (Tassone et al., 2008). However, like the earlier studies, the strategy was not gender-neutral and the first-tier screen was unable to exclude a large percentage of normal female samples, resulting in a significant proportion of normal samples being subjected to the second-tier assay. Subsequently, an improved screening assay involving fluorescent TP-PCR followed by capillary electrophoresis (CE) was reported and used for population screening studies (Tassone et al., 2012).

We recently described a novel single-step screening strategy for rapid identification of *FMR1* CGG repeat expansions based on melt curve analysis (MCA) of TP-PCR products (Teo et al., 2012; Rajan-Babu et al., 2015). This strategy was validated on genotype-known DNA isolated from peripheral blood leukocytes or from cell cultures (Rajan-Babu et al., 2016). To assess the accuracy of the TP-PCR MCA assay when performed on DNA isolated from dried blood spots, we conducted a double-blinded study using dried blood spots obtained from a high risk population, whose genotypes had previously been determined using a different screening method (Winarni et al., 2012). The gender-neutral property of our proposed strategy enables its application to FXS screening in both males and females.

## Materials and Methods

### FXS Reference DNA and Blood Spot Samples

Four genomic DNA samples (NA20232, NA20230, NA20234, and NA20236) derived from lymphoblastoid cell lines obtained from Coriell Cell Repository (CCR; Coriell Institute for Medical Research, Camden, NJ, United States) were used as *FMR1* IM/GZ (46–54 repeats) boundary markers. One hundred and fifty-one previously genotyped dried blood spot samples of individuals with intellectual disability or autism spectrum disorder (75 male and 76 female) were screened in a blinded fashion, with only gender and blood spot identification number (ID) available to the operator. This study was approved by the Institutional Review Boards of the National University of Singapore (B-16-150E) and the Diponegoro University Faculty of Medicine Semarang (PUPT 2016 – Fragile X). The authors assert that all procedures contributing to this work comply with the ethical standards of the relevant national and institutional committees on human experimentation and with the Declaration of Helsinki, as revised in 2013.

### DNA Extraction From Dried Blood Spots

One 6 mm disk was punched out from each dried blood sample spotted onto an FTA card (QIAcard FTA; Qiagen, Valencia, CA, United States). The QIAamp DNA Mini Kit or QIAamp DNA Micro Kit (Qiagen, Hilden, Germany) was used to extract DNA from each disk according to manufacturer’s instructions. The exception when using the QIAamp DNA Micro Kit was the addition of 200 μL of ethanol to the lysate immediately prior to its transfer into the QIAamp MinElute spin column, which significantly improved DNA recovery yield. DNA extracted using QIAamp DNA Mini Kit and QIAamp DNA Micro Kit were eluted in the minimum volume of 50 ul and 20 ul, respectively, according to the manufacturer’s recommendations. DNA samples were quantified using the NanoDrop 1000 (ThermoScientific) and stored at -20°C until use.

### Triplet-Primed PCR (TP-PCR) and Melt Curve Analysis (MCA)

TP-PCR and MCA was performed according to Rajan-Babu et al. (Rajan-Babu et al., 2015) except that final reaction volume was 25 μL and the Rotor-Gene Q High Resolution Melt instrument (RGQ HRM; Qiagen) was used. Each reaction contained 2 μL of dried blood spot-extracted DNA as template, while 50 ng of genomic DNA was used for the CCR IM/GZ control samples. For reactions that did not produce a melt peak, due to low DNA concentration, TP-PCR MCA was repeated with 5 μL or 7.5 μL of extracted DNA. Post-PCR amplicon melting was performed using RGQ HRM, and involved an initial denaturation step at 95°C for 1 min, a hold at 60°C for 1 min, and gradual increase in temperature from 65°C to 95°C in 0.5°C increments with a 5-s hold at each step. Melt peaks were generated by plotting the negative first derivative of change in fluorescence vs. the change in temperature (-dF/dT) against temperature within the HRM module. The melt peak temperature (T_m_) of the last peak before falling to baseline, which corresponds to the temperature exhibiting the greatest change or drop in fluorescence intensity or highest -dF/dT value, was determined. Samples with T_m_ significantly to the right of the upper IM/GZ T_m_ boundary were classified as expansion-positive or expanded, while samples significantly to the left of the lower IM/GZ T_m_ boundary were classified as expansion-negative or non-expanded.

### Fluorescent TP-PCR and Capillary Electrophoresis (CE)

Fluorescent TP-PCR and CE were performed with conditions identical to Rajan-Babu et al. (Rajan-Babu et al., 2015) except that Taq Extender additive was omitted, and either GeneScan^TM^ 500 ROX^TM^ Size Standard (Applied Biosystems – Life Technologies, Carlsbad, CA, United States) or MapMarker1000^®^ (BioVentures, Murfreesboro, TN, United States) was used for internal sizing during CE. Each reaction contained 2 μL of dried blood spot-extracted DNA as template, while 100 ng of genomic DNA was used for the CCR IM/GZ control samples.

### Fluorescent Regular/Repeat-Spanning PCR and CE

Conditions for fluorescent regular/repeat-spanning PCR and CE were similar to those for fluorescent TP-PCR and CE except that the tail and TP primers were replaced with primer 5′-F1 (Teo et al., 2012), 0.5 mM of each dNTP and 0.4 μM of each primer were used in the PCR reaction, and 2 μL of each PCR product was analyzed by CE.

## Results

### Dried Blood Spot DNA Concentrations

Each 6 mm diameter disk of dried blood spot yielded DNA amount ranging from 20 to 1590 ng. Samples with DNA input of at least 20 ng consistently produced clearly analyzable TP-PCR MCA results, although DNA input as low as 3 ng was able to produce melt peaks that were sufficient for analysis.

### TP-PCR MCA Classification of Dried Blood Spot DNA Samples

**FIGURE 1:**
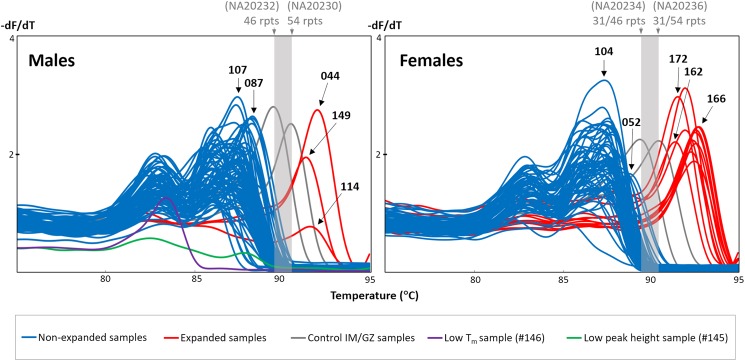
*FMR1* TP-PCR MCA profiles of 75 male and 76 female dried blood spot-extracted DNAs. Gray melt peaks demarcate IM/GZ boundary. Blue melt peaks are classified as non-expanded, while red melt peaks are expanded. Male sample #146 generated a melt peak with unusually low T_m_ (purple), while male sample #145 generated an unusually flat melt peak profile (green).

DNA from dried blood spots of 151 individuals with intellectual disability or autism spectrum disorder were screened for the presence of an expanded *FMR1* CGG repeat using the *FMR1* TP-PCR MCA assay. Among both the male and female groups, a majority of the samples displayed melt peaks (last peak) with T_m_s that were lower than the IM/GZ T_m_ range, indicating that these samples are non-expanded (Figure 1). Three male and 12 female samples generated TP-PCR MCA peaks with T_m_s that were higher than the IM/GZ T_m_ range, and were classified as expanded. There was a clear separation between the melt peaks of the 15 expansion-positive samples and the melt peaks of the other 136 expansion-negative samples.

### Sizing Confirmation of All Expansion-Positive and Selected Expansion-Negative Samples

**FIGURE 2:**
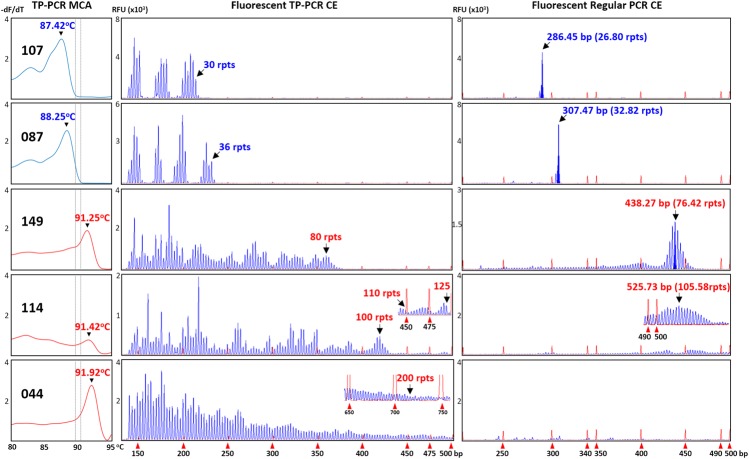
Concordance between TP-PCR MCA, fluorescent TP-PCR CE, and fluorescent regular PCR CE of two representative non-expanded male samples (#107 and #087) and the three expanded male samples (#044, #114, and #149). Gray dotted lines indicate mean T_m_s of IM/GZ boundary. All three assays gave concordant results, except that fluorescent regular PCR CE failed for FM sample #044.

All 15 expansion-positive samples (three male and 12 female), together with 19 representative expansion-negative samples (12 male and seven female) were subjected to sizing confirmation by fluorescent TP-PCR and CE. Fluorescent TP-PCR CE analysis of the three expansion-positive and 12 representative expansion-negative male samples confirmed their expanded and non-expanded classifications, respectively. Fluorescent TP-PCR CE further determined that expanded males #114 and #149 each carry a PM allele while expanded male #044 carries a FM allele (Table 1 and Figure 2).

**Table 1:** Correlation of dried blood spot TP-PCR MCA screen with known genotypes (*n* = 151).

Sample ID	Sex	FXS status	*FMR1* Genotype^a^	TP-PCR MCA^b^	TP-PCR and CE^c^
001	M	NL	29	Neg	n.d.
002	M	NL	31	Neg	n.d.
003	M	NL	30	Neg	n.d.
004	M	NL	37	Neg	n.d.
005	M	NL	35	Neg	n.d.
006	M	NL	39	Neg	n.d.
007	M	NL	30	Neg	n.d.
008	F	NL	30,30	Neg	n.d.
009	F	NL	30,30	Neg	n.d.
010	F	NL	30,30	Neg	n.d.
011	F	NL	30,30	Neg	n.d.
012	M	NL	22	Neg	n.d.
013	M	NL	31	Neg	n.d.
014	M	NL	31	Neg	n.d.
015	M	NL	31	Neg	n.d.
016	M	NL	29	Neg	n.d.
017	F	NL	28,30	Neg	n.d.
018	F	NL	31,31	Neg	n.d.
019	M	NL	31	Neg	n.d.
021	F	NL	31,31	Neg	n.d.
022	M	NL	35	Neg	n.d.
023	F	NL	30,39	Neg	n.d.
024	F	NL	29,32	Neg	n.d.
025	F	NL	31,31	Neg	30/35
026	M	NL	31	Neg	n.d.
027	M	NL	31	Neg	n.d.
028	F	NL	29,29	Neg	n.d.
029	M	NL	36	Neg	n.d.
030	M	NL	29	Neg	n.d.
031	M	NL	30	Neg	n.d.
032	M	NL	38	Neg	n.d.
033	F	NL	30,30	Neg	n.d.
034	M	NL	30	Neg	n.d.
035	M	NL	30	Neg	n.d.
037	M	NL	32	Neg	n.d.
038	M	NL	30	Neg	n.d.
039	F	NL	29,29	Neg	n.d.
040	F	NL	30,30	Neg	n.d.
041	F	NL	30,30	Neg	n.d.
042	M	NL	37	Neg	n.d.
043	F	NL	31,32	Neg	n.d.
**044**	**M**	**FM**	**545**	**Pos**	**> 200**
045	F	NL	28,30	Neg	n.d.
046	M	NL	31	Neg	n.d.
049	F	NL	29,29	Neg	n.d.
051	M	NL	31	Neg	n.d.
052	F	NL	40,40	Neg	39/44
053	F	NL	30,30	Neg	n.d.
054	M	NL	29,51	Neg	n.d.
055	F	NL	29,30	Neg	n.d.
056	M	NL	31	Neg	n.d.
057	F	NL	30,36	Neg	n.d.
058	M	NL	30	Neg	n.d.
059	F	NL	28,28	Neg	n.d.
060	M	NL	29	Neg	n.d.
061	F	NL	20,20	Neg	n.d.
062	F	NL	27,35	Neg	n.d.
063	F	NL	29,29	Neg	n.d.
064	F	NL	29,36	Neg	n.d.
065	M	NL	30	Neg	n.d.
066	M	NL	28	Neg	n.d.
067	F	NL	29,36	Neg	n.d.
068	F	NL	24,26	Neg	n.d.
069	M	NL	26	Neg	n.d.
070	M	NL	33	Neg	n.d.
071	F	NL	26,26	Neg	n.d.
072	M	NL	37	Neg	n.d.
073	M	NL	27	Neg	n.d.
074	M	NL	26	Neg	n.d.
075	F	NL	27,34	Neg	n.d.
076	M	NL	28	Neg	n.d.
077	M	NL	28	Neg	n.d.
078	M	NL	28	Neg	n.d.
079	M	NL	29	Neg	n.d.
080	F	NL	28,28	Neg	n.d.
081	F	NL	30,30	Neg	n.d.
082	M	NL	29	Neg	n.d.
083	M	NL	30	Neg	n.d.
084	F	NL	30,30	Neg	n.d.
085	M	NL	29	Neg	n.d.
086	F	NL	29,29	Neg	n.d.
087	M	NL	36	Neg	36
088	F	NL	28,28	Neg	n.d.
089	F	NL	28,28	Neg	n.d.
090	M	NL	29	Neg	n.d.
091	M	NL	30	Neg	n.d.
092	F	NL	28,28	Neg	n.d.
093	F	NL	27,27	Neg	n.d.
094	F	NL	30,30	Neg	n.d.
095	M	NL	20	Neg	n.d.
096	M	NL	29	Neg	n.d.
097	M	NL	21	Neg	n.d.
098	F	NL	28,35	Neg	n.d.
099	F	NL	28,35	Neg	n.d.
100	F	NL	28,40	Neg	29/41
101	F	NL	27,27	Neg	n.d.
102	M	NL	27	Neg	n.d.
103	M	NL	26	Neg	29
104	F	NL	27,27	Neg	29
105	F	NL	25,25	Neg	n.d.
106	M	NL	28	Neg	n.d.
107	M	NL	30	Neg	30
113	F	NL	29,29	Neg	29/30
**114**	**M**	**PM**	**110**	**Pos**	**∼100,115,125**
125	M	NL	29	Neg	n.d.
126	M	NL	30	Neg	n.d.
127	M	NL	23	Neg	n.d.
128	M	NL	30	Neg	30
131	M	NL	29	Neg	29
133	M	NL	32	Neg	30
136	F	NL	20,28	Neg	n.d.
137	F	NL	15,29	Neg	n.d.
138	M	NL	31	Neg	30
139	F	NL	20,30	Neg	n.d.
140	F	NL	28,28	Neg	29/30
141	F	NL	28,28	Neg	n.d.
142	F	NL	25,30	Neg	n.d.
143	F	NL	29,31	Neg	n.d.
144	F	NL	30,30	Neg	n.d.
145	M	NL	30	Neg	28
146	M	NL	10	Neg	9
147	F	NL	28,28	Neg	29/30
148	M	NL	30	Neg	n.d.
**149**	**M**	**PM**	**83**	**Pos**	**∼80**
150	F	NL	29,29	Neg	n.d.
151	F	NL	22,30	Neg	n.d.
**152**	**F**	**PM**	**29,128**	**Pos**	**29/∼130, > 200**
153	M	NL	29	Neg	29
**154**	**F**	**FM**	**28,110,222,441,504,900**	**Pos**	**29/∼95, > 200**
155	F	NL	29,29	Neg	n.d.
156	F	NL	31,31	Neg	n.d.
**157**	**F**	**FM**	**25,185,324,551,658,800**	**Pos**	**24/> 200**
158	M	NL	30	Neg	n.d.
**159**	**F**	**FM**	**32,423-850**	**Pos**	**32/> 200**
**160**	**F**	**FM**	**24,225**	**Pos**	**24/> 200**
161	F	NL	31,31	Neg	n.d.
**162**	**F**	**PM**	**29,76**	**Pos**	**30/∼75**
163	M	NL	36	Neg	n.d.
164	M	NL	29	Neg	29
**165**	**F**	**FM**	**28,288,532**	**Pos**	**29/> 200**
**166**	**F**	**FM**	**28,223,446,769**	**Pos**	**29/> 200**
167	M	NL	29	Neg	n.d.
168	M	NL	29	Neg	n.d.
**169**	**F**	**PM**	**29,125**	**Pos**	**29/∼130,175**
170	F	NL	30,30	Neg	n.d.
**171**	**F**	**FM**	**34,290**	**Pos**	**34/> 200**
**172**	**F**	**PM**	**30,101**	**Pos**	**30/∼75,100,120**
210	F	NL	28,28	Neg	n.d.
211	M	NL	29	Neg	29
**212**	**F**	**PM**	**30,150**	**Pos**	**30/∼130,135,150,170,180**
213	F	NL	29,29	Neg	n.d.
Total	75M, 76F	**8FM (1M, 7F), 7PM (2M, 5F)** 136 NL (72M, 64F)		**15 Pos (3M, 12F)** 136 Neg (72M, 64F)	

*Male (M) and female (F) samples subjected to sizing confirmation by fluorescent TP-PCR and capillary electrophoresis (CE) are underlined, of which the expansion-positive samples are further indicated in bold. ^*a*^CGG repeat genotypes of all screened samples have been determined previously (Winarni et al., 2012). ^*b*^TP-PCR MCA screening results are shown as Pos (expanded) or Neg (non-expanded). ^*c*^Fluorescent TP-PCR and CE results are shown as number of CGG repeats. Premutation (PM) allele sizes are approximated to the nearest five repeats, and full mutations (FM) are indicated as > 200 repeats. NL, samples with normal allele(s) only; n.d., not determined*.

**FIGURE 3:**
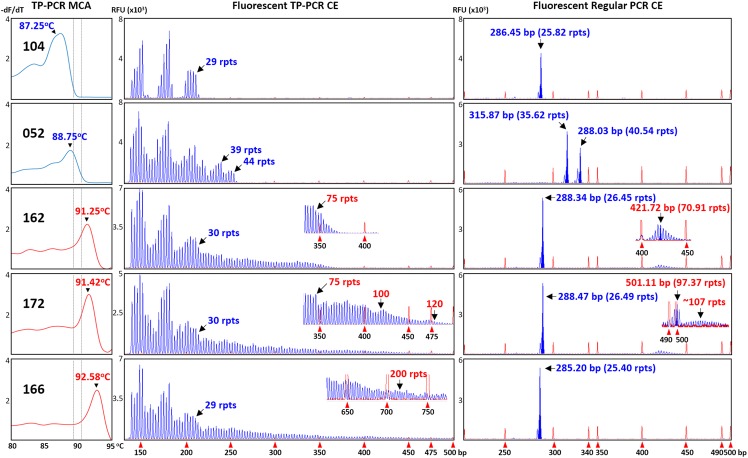
Concordance between TP-PCR MCA, fluorescent TP-PCR CE, and fluorescent regular PCR CE of two representative non-expanded female samples (#052 and #104) and three representative expanded female samples (#162, #166, and #172). All three assays gave concordant results, except that fluorescent regular PCR CE failed to detect the FM allele of sample #166.

The classifications of all 12 expansion-positive and seven representative expansion-negative female samples were also confirmed after fluorescent TP-PCR CE analysis. Fluorescent TP-PCR CE also determined that four expanded females were PM carriers while eight expanded females were FM carriers (Table 1). Representative TP-PCR MCA melt peak profiles and CE electropherograms of expansion-negative and expansion-positive female samples are shown in Figure 3.

To further corroborate the correlations, all 34 samples that were analyzed by fluorescent TP-PCR CE were also subjected to fluorescent regular PCR across the *FMR1* CGG repeat and CE. Fluorescent regular PCR CE results were generally correlated with the TP-PCR MCA classification results, especially for the NL and PM male samples and the PM and some NL female samples. Unlike the TP-PCR MCA screen or fluorescent TP-PCR CE sizing, however, fluorescent regular PCR CE failed to unambiguously detect large expansions from FM male and female samples (Figures 2, 3). Furthermore, compared to repeat sizing by simple peak counting of fluorescent TP-PCR CE products, the calculated repeat size after fluorescent regular PCR CE ([base pair size – 209] ÷ 3) was consistently shorter by ∼4 repeats (Figures 2, 3). This underestimation is likely due to the anomalous mobility of GC-rich amplicons, thus requiring some form of mobility correction, such as the use of standard curves, in order to obtain accurate repeat sizing from fluorescent regular PCR analysis (Biancalana et al., 2015).

**FIGURE 4:**
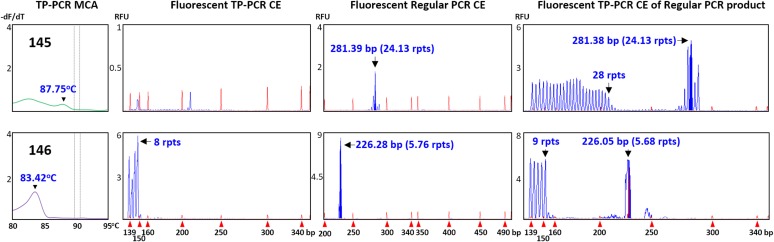
Confirmation of expansion-negative status of samples with unusual results. Top (#145), failed fluorescent TP-PCR CE but successful fluorescent regular PCR CE suggests low DNA concentration. Bottom (#146), fluorescent TP-PCR CE analysis reveals a short CGG repeat. Fluorescent TP-PCR from an aliquot of fluorescent regular PCR product successfully generates a fluorescent TP-PCR CE profile, allowing accurate CGG repeat sizing.

Two of the 12 expansion-negative male samples that underwent sizing confirmation were selected because they generated unexpected TP-PCR MCA melt peak profiles. Sample #145 exhibited an unusually flat melt peak profile while sample #146 produced a distinct melt peak with unusually low T_m_ of 83.42°C (Figure 1). Fluorescent TP-PCR CE indicated that the *FMR1* allele in #146 contains only 8 repeats, which adequately explains its exceptionally low T_m_ in TP-PCR MCA (Figure 4, bottom). In contrast, fluorescent TP-PCR CE of #145 was unsuccessful, suggesting insufficient sample DNA for the reaction, and consistent with the observed flat TP-PCR MCA melt peak profile. However, fluorescent regular PCR CE produced an amplicon peak with a calculated CGG repeat size of 24 before mobility correction. An aliquot of the fluorescent regular PCR product was then used as template for TP-PCR amplification, and this time a fluorescent TP-PCR peak pattern was observed, indicating that the *FMR1* allele in #145 contains 28 uninterrupted CGG repeats (Figure 4, top).

### Reproducibility of Sample Classification From Dried Blood Spot-Derived DNA

**FIGURE 5:**
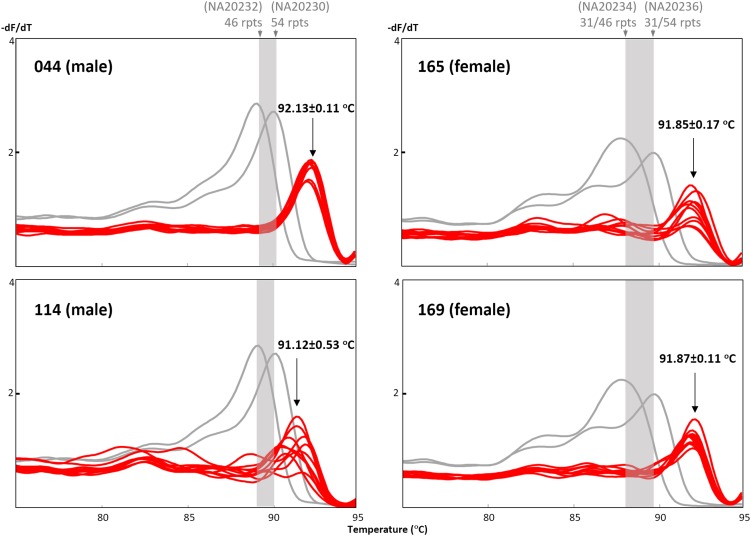
Reproducibility of the TP-PCR MCA classification from dried blood spot-derived DNA. Samples #044 and #169, and to a lesser extent sample #165, generated reproducible and distinct melt peak profiles as compared to sample #114.

Two expansion-positive male and two expansion-positive female dried blood spots were selected for re-extraction using a separate punch from the same blood spot, and DNAs were eluted in 25 μL and re-analyzed in 10 replicates to test the reproducibility of the TP-PCR MCA assay. In the initial screen of 151 samples, samples #044 and #169 produced good melt peak profiles, sample #165 produced a slightly poorer melt peak profile, while sample #114 displayed the flattest melt peak profile. For each sample, the 10 replicates displayed melt peak temperatures consistent with expanded status, although T_m_ variation across replicates differed among samples, with samples #044 and #169 having the lowest standard deviation (SD) of 0.11°C, sample #165 having an intermediate SD of 0.17°C, and sample #114 having the highest SD of 0.53°C (Figure 5). Sample #114 had the lowest DNA concentration of ∼8 ng/μL, whereas the DNA concentrations of the other three samples ranged from ∼26–30 ng/μL. These results suggest that the TP-PCR MCA assay generates reproducible results from dried blood spot-derived DNA generally, although DNA template amount may play a role in T_m_ accuracy.

### Comparison With Sample Genotypes Obtained From Previous Study

**FIGURE 6:**
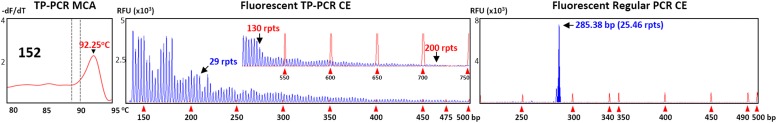
Premutation/full mutation mosaisicm in female sample #152. Fluorescent TP-PCR CE indicates the presence of a ∼130-repeat PM allele, and a FM allele as evidenced by the presence of fluorescent peaks extending beyond 200-repeats.

Screening results of all 151 samples were checked against their previously determined genotypes (Winarni et al., 2012). All 136 expansion-negative samples, and all 15 expansion-positive samples, were correctly classified (Table 1). Since all expansion-positive samples in the screen were followed up by fluorescent TP-PCR CE sizing, their genotypes could also be compared to their previously determined genotypes. All sample genotypes were concordant except for a minor difference in one sample which had been genotyped as a PM female, but fluorescent TP-PCR CE detected evidence of mosaicism for PM and FM alleles (#152) (Figure 6). This genotyping difference, however, does not alter the fact that the TP-PCR MCA screen correctly classified sample #152 as expanded, although further testing will be required to confirm its exact expansion and disease status.

## Discussion

We recently developed a rapid and cost-effective alternative for FXS screening, using a combinatory approach of TP-PCR and MCA (Teo et al., 2012; Rajan-Babu et al., 2015), and also performed a technical validation of the TP-PCR MCA assay to identify optimum performance parameters (Rajan-Babu et al., 2016). We have now demonstrated that this assay is equally accurate when applied to dried blood spot-derived DNA, which is the most readily available and stable DNA source in the context of large-scale or population-based screening, such as newborn screening.

As this assay is optimized to detect expanded alleles regardless of gender, separate analysis and interpretation by gender is unnecessary, hence saving precious time in the screening process. Additionally, confounding factors such as karyotype abnormalities (such as XXX, XXY, and XXYY) will not adversely affect the ability of this assay to detect an *FMR1* expansion when it is present (Biancalana et al., 2015).

Population-screening approaches employing a first-tier regular PCR are inefficient as 43 out of 64 normal females (∼67%) in our test cohort require second-tier CGG-repeat sizing. This is because normal females are excluded only when two normal alleles are detected, and homozygous normal females cannot be distinguished from the much rarer (< 0.1%) affected females carrying a non-amplifiable FM allele. In contrast, the TP-PCR MCA first-tier screening assay identified all 15 expanded male and female samples out of the 151 samples screened (∼10% of total sample count), with no false-positive samples reflexed to CGG-repeat sizing. We also demonstrated that the results generated by this assay are highly reproducible even from dried blood spot-derived DNA, with T_m_s of replicate samples showing minimal deviation.

Additionally, TP-PCR MCA can correctly detect expansions even when DNA concentrations are as low as 1.5 ng/μL. Furthermore, despite the variability of the melt peak profiles generated when the quality of the dried blood spot-derived DNA was sub-optimal, correct expansion classification was not adversely affected. Moreover, while sample #145 (1.0 ng/μL) did not generate a good melt peak profile, the melt peak observed was sufficient for classification as non-expanded after the volume of DNA template was increased to 7.5 μL. Therefore, TP-PCR MCA is very sensitive for screening of FXS, and should be preferred over cytogenetics even in developing countries which lack advanced molecular diagnostic facilities, since this method uses relatively inexpensive equipment.

As a first-tier screen, TP-PCR MCA does not determine CGG-repeat size. Instead, it can be observed that alleles of increasing repeat size will generally have a correspondingly increased T_m_. i.e., T_m_(normal) < T_m_(premutation) < T_m_(full mutation). For the application of TP-PCR MCA in the context of population screening, suitable internal controls that demarcate the IM/GZ boundary are essential in each experiment. To allow for a gender-neutral screen, inclusion of all four genomic DNAs (NA20230, NA20232, NA20234, and NA20236) is advisable. Rapid identification of samples suspected to carry expanded alleles would be achieved simply though observation of melt peak T_m_ position relative to the internal controls. All screen positive samples, as well as any indeterminate/ambiguous samples, should be subjected to CGG-repeat sizing for confirmation/verification of expansion status. Since the TP-PCR MCA screen will also detect PM carriers, who are not FXS-affected but are at risk of developing *FMR1*-related late-onset conditions, genetic counseling should be made available for families of carriers or affected individuals who are identified from the screen. A TP-PCR MCA based screening approach will be very useful for newborn screening purposes as it enables identification of *FMR1* CGG repeat expansion in a single step from both males and females (Tassone, 2014). Early identification will also be extremely beneficial for targeted treatment that has to be executed early in life (Ligsay et al., 2017).

## Author Contributions

SC conceptualized and coordinated the project and experimental design and revised the manuscript. VT conducted the experiments, data analysis and interpretation and wrote the manuscript. ML conducted preliminary experiments and verified the data and revised the manuscript. SF and TW coordinated patient care and dried blood spot collection and revised the manuscript.

## Conflict of Interest Statement

SC is an inventor of the TP-PCR and melt curve analysis strategy described in the manuscript (United States Patent No. US 9,365,892 B2). The remaining authors declare that the research was conducted in the absence of any commercial or financial relationships that could be construed as a potential conflict of interest.
